# Genetics of Obesity: What have we Learned?

**DOI:** 10.2174/138920211795677895

**Published:** 2011-05

**Authors:** Hélène Choquet, David Meyre

**Affiliations:** 1 Ernest Gallo Clinic and Research Center, Department of Neurology, University of California, San Francisco, Emeryville, California 94608, USA; 2 Departments of Clinical Epidemiology and Biostatistics, McMaster University, Hamilton, Ontario L8N 3Z5, Canada

**Keywords:** Biologic pathways, disease prediction, food intake, gene x environment interactions, genetic continuum, Mendelian randomization, obesity, positive selection.

## Abstract

Candidate gene and genome-wide association studies have led to the discovery of nine loci involved in Mendelian forms of obesity and 58 loci contributing to polygenic obesity. These loci explain a small fraction of the heritability for obesity and many genes remain to be discovered. However, efforts in obesity gene identification greatly modified our understanding of this disorder. In this review, we propose an overlook of major lessons learned from 15 years of research in the field of genetics and obesity. We comment on the existence of the genetic continuum between monogenic and polygenic forms of obesity that pinpoints the role of genes involved in the central regulation of food intake and genetic predisposition to obesity. We explain how the identification of novel obesity predisposing genes has clarified unsuspected biological pathways involved in the control of energy balance that have helped to understand past human history and to explore causality in epidemiology. We provide evidence that obesity predisposing genes interact with the environment and influence the response to treatment relevant to disease prediction.

## INTRODUCTION

In 2001, six genes were linked to monogenic human obesity and no common variants were reproducibly associated with polygenic obesity. By 2008, progress in the field led to the discovery of eight monogenic genes and four polygenic genes (*FTO, PCSK1, MC4R, CTNNBL1*) from associated studies at the genome-wide level of significance. The recent emergence of the genome-wide association studies (GWAS) has led to further breakthroughs in gene identification and now nine loci are recognized to be involved in Mendelian forms of obesity along with 58 loci contributing to polygenic obesity. In this review, we will discuss what we have learned from this recent progress in elucidating the molecular basis of obesity. We propose an overlook of major lessons learned from 15 years of research in the field of the genetics and obesity.

## GENETICS OF OBESITY: LESSONS LEARNED

### A Continuum between Monogenic and Polygenic Obesity

A striking observation is the existence of a partially overlapping continuum between monogenic and polygenic forms of obesity. Currently, four genes (*MC4R, PCSK1, POMC *and* BDNF*) have been involved in the two conditions, and this list is likely to grow in the upcoming years. This continuum is not specific to obesity, since one-fifth of the loci that were found to be associated with complex disease traits include a gene that is mutated in a corresponding single-gene disorder [1]. The case of *MC4R* is illustrative of this point of view. Whereas more than 150 loss-of-function coding mutations have been associated with monogenic obesity [2], two infrequent gain-of-function coding polymorphisms (V103I and I251L) have been associated with the protection from obesity [3, 4]. Furthermore, a SNP located 188 kb downstream of the *MC4R* coding sequence has been associated with a modest increase in the risk for obesity [5]. 

Dickson *et al*. [6] have recently proposed the synthetic association hypothesis: GWAS signals of common non-functional SNPs outside of coding regions may be the result of a combination of rare/coding functional variants with stronger effects given that these rare variants arose on a haplotype which is tagged by the common SNP. This hypothesis, if true, may explain why some genes are associated with monogenic and polygenic forms of the same disease. A careful investigation of *MC4R* has therefore invalidated the synthetic association hypothesis at this locus, and supports the concept of an independent contribution of both rare and common variants at the same locus for obesity risk [7]. 

Another lesson of the observed continuum between monogenic and polygenic forms of obesity is that GWAS-derived novel loci should be considered as highly relevant candidate genes for monogenic obesity, especially if additional arguments in humans or animal models strengthen the candidacy of the gene. The *SH2B1* gene is for instance an interesting candidate as SNPs at the *SH2B1* locus are associated with BMI by GWAS [8], rare deletions including *SH2B1* are associated with a Mendelian form of obesity [9, 10] and inactivation of SH2B1 in mice leads to hyperphagia, leptin resistance and obesity [11]. However, notable exceptions have been reported for other promising candidate genes. For example, *FTO* is the major contributor to polygenic obesity [12] and mice down or over-expressing *FTO* are resistant or prone to develop obesity [13, 14]. However, heterozygous loss-of-function mutations in *FTO* are found in both lean and obese subjects and do not contribute to monogenic obesity [15].

### Obesity is an Inherited Disorder of Central Regulation of Food Intake 

Defects in eight genes involved in the neuronal differentiation of the paraventricular nucleus and in the leptin/melanocortin pathway, have been shown to lead to human monogenic obesity with hyperphagia as a common feature [16]. Recent progress in the elucidation of polygenic predisposition to obesity also points to a key role of the central nervous system in body weight regulation [17]. 

The association of the two major contributors to polygenic obesity (SNP rs17782313 near *MC4R* and SNP rs1421085 / rs9939609 in *FTO*) [5, 18, 19] with food intake / food behavior-related endophenotypes has been well documented in the literature. The obesity predisposing *FTO* variant was associated with increased total and fat dietary intake in children [20, 21] as well as in adults [22]. The obesity risk variant was also associated with diminished satiety and / or increased feeling of hunger in children [23] and in adults [24]. The obesity predisposing SNP variant near *MC4R* was associated with increased feeling of hunger [25, 26], increased snacking [25], decreased satiety [26], and increased total, fat and protein energy intake [25, 27], the effects of the variant on food-related parameters being observed both in children and adults. Bauer *et al*. [28] recently reported evidence for an association of additional obesity genes recently identified by GWAS (*SH2B1, KCTD15, MTCH2, NEGR1, BDNF*) with dietary intake and nutrient-specific food preference. 

The genetic dissection of monogenic and polygenic forms of obesity delineate it as an inherited disorder of central regulation of food intake [16]. This is in line with the fact that food intake-related parameters are heritable [29] and are strongly correlated to body mass index [30]. 

### Gene Identification Illuminates New Pathways Involved in Energy Balance

A primary goal of human genetic agnostic approaches such as GWAS is to improve our understanding of the biologic pathways underlying polygenic diseases and traits [1]. A majority of obesity loci identified by GWAS studies do not harbor genes with clear connections to the biology of body weight regulation [12], reflecting our limited understanding of the biology of obesity in contrast with other complex traits (such as autoimmune diseases or lipid levels). Recent progress in genetic dissection of obesity predisposition provides the opportunity to explore novel and sometimes unsuspected pathways related to this condition. 

The *FTO* story constitutes a textbook case: the *FTO* gene has been cloned in mice in 1999 [31] but the “buzz” around this gene started after the publication of two seminal genetic studies demonstrating a link between *FTO* common gene variation and human obesity in 2007 (at this time, *FTO* was a gene of unknown function in an unknown pathway) [18, 19]. More than 400 articles have been published since increasing our understanding of the mechanisms linking this gene to the pathophysiology of obesity. Follow-up studies have confirmed the association between *FTO* and obesity-related phenotypes not only in populations of European ancestry but also in African, Asian, South Asian, South American and Pima Indian populations [32-36]. 

Complete FTO deficiency in humans is associated with an autosomal-recessive lethal syndrome including growth retardation, multiple malformations and premature death, indicating that FTO is essential for normal development of the central nervous and cardiovascular systems in human [37]. Loss of one functional copy of *FTO* in humans was not associated with a specific phenotype, and heterozygous loss-of-function mutations are found both in lean and in obese subjects [15]. Complete or partial inactivation of the *Fto* gene in mice protects from obesity [13, 38] whereas over-expression of *Fto* in mice increases food intake and results in obesity [14]. These data have provided direct functional evidence that *FTO* is a causal gene underlying obesity, and suggest the intronic variant in *FTO* may increase obesity risk in humans through *FTO* gain of expression. Expression studies in wild-type rodents have shown that FTO is highly expressed in the hypothalamus and is regulated by feeding and fasting [39, 40]. Over- or down-expression of *Fto* in the hypothalamus modulates food intake in mice possibly through the leptin / STAT3 signalling pathway [40, 41]. 

Bioinformatics, *in vitro* and crystallography studies have shown that *FTO* is a single-stranded DNA demethylase and is involved in nucleic acid repair or modification processes [39, 42]. The link between this genes’ ability to modify nucleic acids and body weight regulation may be puzzling, but may relate to epigenetic processes. FTO has been proposed as a transcriptional coactivator that enhances the transactivation potential of the CCAAT / enhancer binding proteins (C/EBPs) from unmethylated as well as methylation-inhibited promoters, suggesting a role in the epigenetic regulation of the development and maintenance of fat tissue [43]. In line with this hypothesis the fact that the *FTO* intronic SNP is associated with a distinct methylation pattern of a 7.7 kb region at the *FTO* locus, that includes a highly conserved non-coding element validated as a long-range enhancer [44]. 

The study of *FTO* illustrates how human genetic “hypothesis free” approaches can be a catalyst to approaches in functional genomics and the same integrative approach can be applied to other obesity-associated genes markedly increasing our understanding of the physiology of obesity in the upcoming years. 

### Obesity Genes to Explore Causality in Epidemiology: The Mendelian Randomization Approach 

Spurious associations in observational epidemiological studies are commonly caused by confounders due to social, behavioral, or environmental factors and can therefore be difficult to control. They may also be due to reverse causation, in which the phenotypic outcome subsequently influences an environmental exposure such that it is wrongly implicated in the pathogenesis. Genetic epidemiology can be used to uncover more thoroughly and more accurately causal factors underlying common diseases or complex traits. The epidemiologic approach with the most promise is often referred to as Mendelian randomization [45]. The principle is to use genetic variation as a randomly redistributed variable among populations to control for unobserved confounding variables in an observational setting [45]. 

This approach has been successfully used in the obesity arena following the identification of *FTO* as a major contributor to polygenic variation [18]. The Frayling *et al.* [18] seminal study represents in fact a good example of Mendelian randomization since a BMI-dependent association between *FTO* and type 2 diabetes mellitus (T2D) has been observed, suggestive of a causative relationship between weight gain and subsequent T2D development. The same approach, when applied to 12 obesity gene variants, confirmed that the genetic predisposition to obesity leads to an increased risk of developing type 2 diabetes, which is completely mediated by its effect on BMI [46]. The *FTO* genotype has also been used to confirm the findings of observational epidemiology and a causal relationship between BMI increase and altered glucose [47], insulin resistance [48], lipid [47] and blood pressure values [49]. The causal association between increased BMI and increased level of inflammation [50] or increased bone mass [51] has also been confirmed, and the use of *FTO* SNP as a randomly redistributed variable strengthened the evidence of a causal link between a BMI increase and an increased risk of atherosclerosis [52], cardiovascular diseases [53], endometrial or kidney cancers [54, 55] and a decreased risk of lung or prostate cancers [54, 56]. 

### Genes are Useful to Understand Past Human History

The human genome contains hundreds of regions whose pattern of genetic diversity indicate recent positive natural selection (positive natural selection is the force that drives the increase in prevalence of advantageous traits like *de novo* mutations) [57]. Adaptation to new environments, infectious diseases and changes in diet may explain why certain mutations have been positively selected in human populations [58]. The transition to agriculture has introduced new adaptive pressures that shaped our genome to an increased fat storage efficiency including exposure to regular famine, adaptation to a variety of local niches favoring population-specific adaptations and the development of social hierarchies which predispose to differential exposure to environmental pressures [59]. The “thrifty genotype” hypothesis, proposed by Neel in 1962 [60], was recently confirmed for several obesity genes that show evidence of positive selection across human history. For example, the rs4988235 functional variant in the lactase (*LCT*) gene confers lactase persistence and carriers of at least one T allele, are able to digest the milk sugar lactose across their life span (the activity of the lactase enzyme in intestinal cells declines during childhood in non-carriers) [61, 62]. The selective advantage of lactase persistence in milk-producing dairy farming populations has induced positive selection signatures regionally for the *LCT* rs4988235 T variant strongly related to events of cattle domestication [63, 64]. A North to South gradient has been observed for the LCT rs4988235 SNP in Europe [63] as well as local geographic population substructures among provinces in the United Kingdom [65]. In line with its proposed selective advantage, the *LCT* rs4988235 T variant has been consistently associated with higher milk consumption [66] and with higher body mass index [67] in European populations.

Interestingly, three functional coding non-synonymous variants in the *LEPR* (Lys109Arg), *ADRB3* (Trp64Arg) and *BDNF* (Val66Met) genes previously associated with BMI [8, 68, 69], harbor patterns of strong positive selection in population genetic studies [70-72]. Recently, analysis of GWAS-derived obesity gene variants provided evidence of positive natural selection at the *FTO*, *NEGR1*, *SH2B1* and *FAIM2* loci [73, 74]. 

The evolutionary history of the *MC4R* obesity locus has been well documented in the literature. The melanocortin 4 receptor coding sequence has been remarkably conserved in structure and pharmacology for more than 400 million years, implying that this receptor participated in vital physiological functions early in vertebrate evolution [75]. There is a significant paucity of diversity at the *MC4R* gene in humans in comparison with primates [76]. The coding region of *MC4R* has been subject to high levels of continuous purifying selection that increased threefold during primate evolution [76]. Finally, there is a tendency for non-synonymous mutations that impact *MC4R* function to be located at amino acid positions that are highly conserved during the 450 million years of *MC4R* evolution in vertebrates and subject to very strong purifying selection [76].

### Genes Interacting with Environments 

As trends over the past several decades suggest an environmental influence on BMI, many researchers have focused on the identification of specific environmental factors that interact with genetic predisposition to obesity. They based their investigations on epidemiological data showing that physical activity, diet, educational status, age, gender and ethnicity among others modulate the risk for obesity [77]. 

Recent literature provides firm evidence that genetic susceptibility to obesity can be blunted in part through physical activity. Thirteen independent studies reported an interaction between the *FTO* obesity risk genotype and physical activity on BMI variation or obesity risk including adults as well as adolescents [78-81]. Similar results were obtained for a genetic predisposition score combining the information of 12 obesity-associated SNPs, and a high level of physical activity associated with a 40% reduction in the genetic predisposition to common obesity [82]. 

There is also growing evidence that dietary habits interact with genes to modulate predisposition to obesity. Three studies suggest that a high fat diet can amplify the effect of the *FTO* genotype on obesity risk [79-81]. An interaction between the Apolipoprotein A-II (*APOA2*) -265T>C SNP and high-saturated fat in relation to BMI and obesity has been reported in five independent populations [83, 84]. Interestingly, this SNP was not identified by recent GWAS approaches, suggesting that some associations restricted to specific environments may be missed in global analyses. 

Epidemiological studies have shown that people with a low level of education are more likely to develop obesity [85]. However, very few studies have investigated the impact of genes on the association between education and obesity-related variables. This well-established negative association between BMI and educational status was not found in *MC4R* loss-of function mutation carriers, although a significant relationship was seen in *MC4R* non-mutation carriers of the corresponding pedigrees [2]. These results suggest that a high level of education has no protective effect on obesity risk in presence of *MC4R* pathogenic mutations. On the contrary, a significant gene x education interaction has been found for the intron 1 variant in FTO, the significant effect of the SNP on BMI and obesity risk being restricted to subjects with no university education [86]. 

Age-dependent genetic associations have been described both in the context of monogenic and polygenic obesity. An age-dependent penetrance of *MC4R* pathogenic and monogenic mutations on obesity has been found in multigenerational pedigrees, the effect of mutations on the obesity phenotype being amplified by the development of an "obesogenic" environment [2]. The longitudinal study of adult *MC4R* mutation carriers showed an increasing age-dependent penetrance (37% at 20 years versus 60% at >40 years) [2]. The life-course analysis of the intronic *FTO* gene variant and body mass index in independent longitudinal studies indicates that most of the effect of the SNP on BMI gain occurs during childhood, adolescence and young adulthood [87-89]. 

Gender can be assimilated as a specific environmental condition. Females are at higher risk of developing morbid obesity than males [90]. These discrepancies could be explained in part by female-specific genetic associations or by stronger effect sizes of genetic variants in females. This was observed for the carriers of *MC4R* pathogenic monogenic mutations since BMI was about twice as strong in females than in males [2, 91]. The effect of the functional polymorphism R125W polymorphism in TBC1 domain family member 1 (*TBC1D1*) gene on severe obesity risk was restricted to females in French and US populations [92, 93]. Seven out of 14 loci convincingly associated with waist to hip ratio exhibited marked sexual dimorphism, all with a stronger effect on the phenotype in women than men [94, 95].

Ethnicity can be considered as an environmental factor that affects the genetic susceptibility to obesity. A convincing example of ethnic-specific association with obesity has been reported for the *SIM1* gene. Variants in intronic regions of *SIM1* were strongly associated with BMI and obesity risk (P = 4 x 10^-7^) in Pima Indians contrarily to French Europeans for which a major contribution of *SIM1* common variants in polygenic obesity susceptibility was excluded [96, 97]. A functional coding variant (W64R) [98] in the *ADRB3* gene has also been convincingly associated with BMI in East Asian but not in European subjects in a large meta-analysis of 44,833 subjects [69], the effect of the R64 allele on BMI increase being four-fold higher in Asian than in European subjects. More recently, a SNP (rs2074356) in the 24^th^ intron of the C12orf51 transcript has been strongly associated with waist to hip ratio (P = 7.8 x 10^-12^) in the Korean population [99]. This variant has not been identified in a large GWAS meta-analysis for waist to hip ratio conducted in 77,167 individuals of European ancestry [95], suggesting an ethnic-specific association at this locus. These studies highlight the complex interplay between genetic susceptibility to obesity and environment. 

### Genes Influencing Response to Treatment

To date, three main therapeutic options are proposed to treat obesity: lifestyle intervention, pharmacotherapy and bariatric surgery. The aim of these therapeutics for obesity are to lose weight and maintain this weight-loss on the long term and attenuate co-morbidities related to obesity. There is growing evidence that genetic factors not only predispose to weight gain and development of obesity, but also modulate the response to therapeutic intervention in terms of weight loss.

#### Lifestyle Modifications

Individuals with* MC4R* or *POMC* monogenic conditions respond well to hypocaloric dietary or multidisciplinary (exercise, behavior, nutrition therapy) interventions as do non-monogenic obese subjects [100, 101] but *MC4R* individuals fail to maintain weight loss after intervention [101]. The major gene variant contributing to polygenic obesity *FTO* does not modify the response to lifestyle intervention in terms of weight loss [102], but may interact with specific components of the lifestyle intervention program like the type of diet proposed during the caloric restriction program (high-fat, low-fat, Mediterranean diets) [103, 104] or with physical activity [105] to modulate weight loss. 

#### Pharmacotherapy

To date, only one successful personalized medicine approach which is based on a genetic diagnosis has been reported in the literature in the context of obesity: individuals with congenital leptin deficiency can be treated with daily injections of recombinant human leptin, which reverses the obesity and associated phenotypic abnormalities [106]. Leptin administration dramatically reduces food intake, fat mass, hyperinsulinemia, and hyperlipidemia, restores normal pubertal development, endocrine and immune function and increases performances in many neurocognitive domains [107]. Individuals with complete leptin deficiency are extremely rare (14 are reported so far worldwide) but peripheral leptin supplementation may also be extended to a numerically significant group of obese subjects with partial leptin deficiency, on the observation that peripheral leptin supplementation induces significant weight loss in those with low levels of leptin [108]. In addition, chemical chaperones and pharmacological agonists efficiently restore cell surface expression and endogenous agonist response of mutated melanocortin 4 receptors [109, 110], but *in vivo* beneficial effects in MC4R deficient monogenic patients remain to be demonstrated.

To date, the two main anti-obesity drugs used are orlistat and sibutramine (a saturated derivative of lipstatin and a serotonin-norepinephrine reuptake inhibitor, respectively). The guanine nucleotide binding protein beta polypeptide 3 (*GNB3*) gene C825T polymorphism is highly predictive for the identification of obese individuals who will benefit from sibutramine treatment. Thus, three independent pharmacogenetic studies have shown an association between the *GNB3 *C825T polymorphism and weight loss induced by sibutramine [111-113]. Interestingly, this locus has not been identified by recent GWAS on obesity-related traits, suggesting that the genes associated with BMI variation and obesity risk may be at least partly different from genes involved in therapeutic response in terms of weight loss. 

#### Bariatric Surgery

Bariatric surgery is the most effective long-term treatment for severe obesity, reducing obesity-associated co-morbidities but the mechanisms of weight loss after bariatric surgery and the role of central energy homeostatic pathways in this weight loss process are not well understood. Two recent studies assessed the response to bariatric surgery of *MC4R* monogenic mutation carriers [114, 115]. The Rouxen-Y gastric bypass surgery was associated with a similar percentage of excess weight loss in four heterozygous *MC4R* mutation carriers and in matched *MC4R* mutation non-carrier obese controls [115]. On the contrary, an adolescent with complete MC4R deficiency underwent laparoscopic adjustable gastric banding at 18 years of age which resulted in an initial, but not long-term weight loss [114]. These preliminary results need to be confirmed in larger studies, but logically suggest that diversionary operations, which are more invasive, efficiently improve the neuro-hormonal control of satiety better than gastric banding procedures, and are therefore indicated in monogenic hyperphagic subjects. 

In the Swedish obesity intervention study, the *FTO *obesity predisposing allele carriers lost 3kg less than common allele homozygotes after obesity surgery, and this association was restricted to those undergoing banding surgery but was not significant in the gastric bypass operated subjects [116]. In a cohort of 1,001 severely obese subjects who underwent gastric bypass surgery, an allelic risk score combining the genetic information of four obesity-associated SNPs was significantly associated with postoperative weight loss trajectories [117]. Thus, obesity predisposing genes modulate the response to therapeutic options in terms of weight loss suggesting that genetic diagnosis combined with a genomic personalized medicine approach is a plausible strategy in order to design and implement the most suitable treatment and to achieve higher rates of therapeutic success.

### Obesity Genes and Disease Prediction

Traditional approaches for the management of overweight and obesity have proven poor long term efficacy and obesity surgery is an efficient but invasive procedure. Prevention may therefore be considered as a promising strategy to face the obesity epidemic. In that context, the use of genetic information in clinical practice to predict individuals at high risk early in life and before the development of the disease remains the 'Holy Grail' for many geneticists [118]. Is the current knowledge about obesity genetics, sufficient to envisage such translational medicine applications? 

#### Common Genetic Variants: Still Few Informative 

GWAS allowed the identification of 36 polymorphisms robustly associated with BMI. However, identified variants have small effect sizes and collectively explain 1.45% of the variance in BMI (0.34% explained by the SNP in intron 1 of *FTO* alone) [12]. Therefore, it is not surprising that the combined information of 12-20 obesity predisposing SNPs provides only a slight increase in the ability to predict obesity in comparison with conventional nongenetic risk factors and has no clinical utility [119]. Risk prediction using GWAS remain conceivable despite the fact that individual effect sizes of variants associated with the phenotype are mostly small. In fact, it seems that many disease-associated variants are not yet identified prospects for risk prediction, but may improve if more disease predisposing variants are included in the models [120]. New iterative algorithms have been recently proposed to make better use of the whole-genome SNP information to improve the performance of disease risk assessment by utilizing a larger number of SNPs than those which reach genome-wide significance [120, 121]. 

#### Monogenic Genes may Explain a Non-Negligible Fraction of Obesity 

The cumulative prevalence of monogenic obesity elucidated by the eight currently known genes and the 16p11.2 deletion has not been evaluated in a randomly ascertained cohort of obese subjects to date, but may be estimated between 5 and 10 %. These results re-emphasize the importance of monogenic obesity in elucidating the heritability of obesity, and Mendelian forms of obesity may provide a non-negligible predictive value in classifying young subjects at high risk for the development of childhood obesity, as deleterious coding mutations or chromosomal aberrations in these genes / regions induce highly penetrant forms of obesity. subjects carrying these mutations, present specific features according to the impaired gene (such as a low level of circulating leptin despite severe obesity, a susceptibility to infections, intestinal dysfunction, reactive hypoglycaemia, red hair and pale skin, adrenal insufficiency) that can guide gene sequencing approaches. Early diagnosis is fundamental for personalized prevention and effective therapeutic management, and in young non-obese individuals carrying *MC4R* monogenic mutations, an appropriate medical follow-up to prevent or at least delay the onset of obesity [122].

As hyperphagia is a common feature of monogenic obesity, the most effective preventive strategy may be stringent restriction of food access. This will require training and active participation of the parents and care providers and the identification of critical environmental components (physical activity, rural / urban environment, dietary profile, family structure, socioeconomic status, social network, psychosocial stress) that modulate the penetrance of obesity associated with pathogenic mutations in order to avoid unhealthy environments for these subjects. 

In conclusion, we have demonstrated that 15 years of gene identification efforts have considerably modified our understanding of the biology of obesity. Promising approaches such as whole-exome and ultimately whole-genome sequencing have the potential to lead to an exhaustive map of obesity predisposing genes in the near future. Recent gene identification efforts have provided a more comprehensive picture of the biological mechanisms involved in the development of obesity and we feel that this information can be meaningful not only for scientists and clinicians but for a more general audience. For instance, the recent discoveries in genetics have found that people differ in their perceptions of hunger and satiety on a genetic basis and that predisposed subgroups of the population may be particularly vulnerable to obesity in “obesogenic” societies with unlimited access to food. This notion must lead to a more open attitude toward obese people and a reduction in discrimination against them [123], it is clear that obesity cannot be considered as a consequence only of indolence or lack of will, as often thought in our societies. In the long term, we are confident that progress in genetics will help to develop useful diagnostic and predictive tests and design new treatments. 
